# Surveillance of post-cataract endophthalmitis at a tertiary referral center: a 10-year critical evaluation

**DOI:** 10.1186/s40942-021-00280-1

**Published:** 2021-02-16

**Authors:** Juliana Mika Kato, Tatiana Tanaka, Luiza Manhezi Shin de Oliveira, Maura Salaroli de Oliveira, Flavia Rossi, Mauro Goldbaum, Sergio Luis Gianotti Pimentel, João Nóbrega de Almeida Junior, Joyce Hisae Yamamoto

**Affiliations:** 1 Department of Ophthalmology-LIM33, Hospital das Clinicas HCFMUSP, Faculdade de Medicina, Universidade de São Paulo, 255 Av. Dr. Enéas Carvalho de Aguiar, São Paulo, Brazil; 2 Division of Infectious and Parasitic Diseases, Hospital das Clinicas HCFMUSP, Faculdade de Medicina, Universidade de São Paulo, São Paulo, Brazil; 3 Central Laboratory Division-LIM03, Hospital das Clinicas HCFMUSP, Faculdade de Medicina, Universidade de São Paulo, São Paulo, Brazil; 4 Institute of Tropical Medicine-LIM53, University of São Paulo (USP), São Paulo, Brazil

**Keywords:** Endophthalmitis, Cataract surgery, Antibiotic resistance

## Abstract

**Background:**

Acute post-cataract endophthalmitis (APE) is a rare complication potentially causing irreversible visual loss. A 10-year study of APE was conducted to determine its incidence, microbiological spectra and antibiotic resistance profile of APE-related pathogens at a major tertiary referral center in Brazil.

**Methods:**

APE cases reported between January 2010 and December 2019 were included. Phacoemulsification and extracapsular cataract techniques were eligible; combined procedures, traumatic and congenital cataract were excluded. Vitreous samples were cultured and antimicrobial resistance was compared for the periods of 2010–2014 and 2015–2019. The results were analyzed with Fisher’s exact test.

**Results:**

Our sample consisted of 40,491 cataract surgeries and 51 (0.126%) APE cases. Culture was positive in 35 cases (71.4%), of which 31 (88.6%) Gram-positive, 3 (8.6%) Gram-negative, and 1 (2.9%) fungal. The most frequently isolated organism was *Staphylococcus epidermidis* (n = 17/35, 48.6%), followed by *Staphylococcus aureus* (n = 4/35, 11.4%). From 2010–2014 to 2015–2019, antimicrobial resistance increased against moxifloxacin (11.1–54.5%, *p* = 0.07), ciprofloxacin (54.5–72.7%, *p* = 0.659) and oxacillin (66.7–93.3%, *p* = 0.13).

**Conclusions:**

The observed incidence and microbial spectra were compatible with previous studies. A trend towards growing moxifloxacin and ciprofloxacin resistance was observed. Surveillance remains crucial to prevent treatment failure from antimicrobial resistance.

## Background

According to the World Health Organization, cataract is the leading cause of reversible blindness, affecting about 20 million Americans in 2010 and possibly as many as 50 million by 2050 [1]. Due to population ageing, the proportional demand for cataract surgery is expected to grow in the next few years. However, complications like endophthalmitis can lead to severe visual impairment and even anatomic distortion of the ocular globe. The reported incidence of acute post-cataract endophthalmitis (APE) is declining, with current rates between 0.029 and 0.29% [2–4], though rates may vary from country to country (e.g., rates are low in Sweden [3] and high in some centers in Brazil and the UK) [2, 4]. Recent reports have shown a further decrease of APE rates when antibiotic is injected into the anterior chamber [5, 6]. Many European studies have adopted the use of cefuroxime, while American studies seem to prefer the fourth-generation fluoroquinolones [5, 7].

Currently, APE prevention and management depend on empirical antibiotic therapy. Even when referral and treatment are timely, outcomes remain generally poor. Overall, less than half of the patients achieve final visual acuity of 20/40 after treatment [8, 9]. Visual prognosis depends on acuity at presentation, comorbidities, etiological agent and antibiotic resistance.

Very little has been published in Brazil on APE [2, 10]. A 5-year study comparing patients with and without use of intracameral (IC) antibiotics found an incidence of 0.03% and 0.22%, respectively [10]. Local surveillance of causative agents and microbial spectra can help identify possible trends in antibiotic resistance and improve guidelines for prophylaxis and treatment. Thus, the purpose of this study was to determine the incidence, microbiological spectrum and antibiotic resistance profile of APE-related pathogens at a major tertiary referral center in Brazil and compare trends for the periods 2010–2014 and 2015–2019.

## Methods

 This 10-year retrospective, descriptive, observational study was carried out between January 2010 and December 2019 at a Brazilian tertiary referral center (Hospital das Clinicas HCFMUSP, Faculdade de Medicina, Universidade de Sao Paulo, Sao Paulo, SP) attended only by patients from the public health care system. All cataract surgeries using phacoemulsification and extracapsular techniques were eligible. Presumed traumatic cataract or congenital cataract surgeries and combined procedures (e.g., trabeculectomy and vitreoretinal procedures) were excluded. Information on surgeries was retrieved from the hospital’s database by coding search.

The diagnosis of APE was based on the presence of decreased vision, painful eye, hypopyon, or vitreitis developed within 6 weeks after the surgery. All patients submitted to cataract surgery at our hospital are strongly advised to return in case of symptoms or signs of infection, but even if diagnosed elsewhere APE patients are highly likely to be referred back for management. Moreover, cataract surgeons (residents and cataract fellows) are instructed to notify APE cases to the research team. The records of the identified cases were reviewed with regard to clinical settings, demographics, microbiological findings and antibiotic susceptibility.

As a matter of routine, all patients underwent preoperative eyelid and periocular surface antisepsis with 10% povidone-iodine and instillation of 5% povidone-iodine eye drops prior to draping. IC antibiotic prophylaxis (an uncommon practice at our service) was not performed. Postoperatively, moxifloxacin and steroid eye drops were prescribed every 2 hours for the first two days, then every 4 hours for 1 week. The steroids were gradually reduced until completing a month. Combined eyedrops were provided by the hospital.

Vitreous fluid samples were obtained by tap using a 22-gauge needle attached to a 3–5 mL syringe or by pars plana vitrectomy. To determine the etiology of each case, the samples were sent to the institution’s microbiology lab and either cultured in thioglycolate broth for 5 days at 35 °C (Jan 2010–Dec 2011) or inoculated in pediatric blood culture bottle (BACTEC Plus Aerobic/F, BD diagnosis, USA) and incubated in automated machines for up to 5 days (Jan 2012–Dec 2019), as described elsewhere [11]. Positive samples from either method were seeded onto culture plates (blood sheep and chocolate agar) and incubated for 48 h in a 5% CO_2_ atmosphere, as per standard procedure [12]. Bacterial isolates were identified with either a Vitek 2 automated method (bioMérieux, Marcy-l’Etoile, France) (Jan 2010–Feb 2015) or Vitek MS (bioMérieux, Marcy-l’Etoile, France) (Mar 2015–Dec 2019) [13]. Fungal isolates were identified by macro and micromorphology. Susceptibility testing was done with a Vitek 2 system (bioMérieux, Marcy-l’Etoile, France) and the results were interpreted according to the current Clinical & Laboratory Standards Institute (CLSI) breakpoints [14].

The periods 2010–2014 and 2015–2019 were compared with regard to antimicrobial resistance. The results were then submitted to Fisher’s exact test for trend analysis (SPSS v. 22.0), with the level of statistical significance set at 5% (*p* < 0.05).

## Results

During the 10-year study period, 40,491 cataract surgeries were performed and 51 patients developed APE, leading to an overall APE rate of 0.126% (95% CI 0.075–0.187). The number of cataract surgeries and APE cases and the annual incidence are shown in Table 1. No cases of bilateral APE were observed.

**Table 1:** Annual rate of acute postoperative endophthalmitis (APE) and sample positivity for the identification of etiological agents. Period: 2010–2019

Year	APE cases	Number of cataract surgeries	Annual APE rate (%)	Positive/ analyzed samples (%)
2010	3	3589	0.084	1/3 (33.3)
2011	7	4426	0.158	4/7 (57.1)
2012	7	4118	0.170	5/7 (71.4)
2013	5	5112	0.098	3/5 (60.0)
2014	4	5282	0.076	3/4 (75.0)
2015	2	4193	0.048	0/1 (0)
2016	9	3269	0.275	6/9 (66.7)
2017	5	3338	0.150	5/5 (100)
2018	1	3547	0.028	1/1 (100)
2019	8	3617	0.221	7/7 (100)
Total	51	40,491	0.126	35/49 (71.4)

Our sample of APE patients included 26 men (51%) and 25 women (49%), aged 68.7 ± 11.3 years on average (range 32–87). The mean time from surgery to clinical presentation was 6.0 ± 5.1 days (range 1–21). Thirty-eight patients (74.5%) were submitted to clear corneal incision phacoemulsification while the remaining 13 patients (25.5%) were treated with extracapsular cataract extraction.

Vitreous samples from 49 of the 51 APE cases were cultured, yielding 71.4% positivity (n = 35). The isolates were predominantly Gram-positive bacteria (n = 31, 88.6%), followed by Gram-negative bacteria (n = 3, 8.6%) and fungi (n = 1, 2.9%) (Table 2). The most frequently observed *Staphylococcus* species (n = 24) were *S. epidermidis* (n = 17, 48.6%), *S. aureus* (n = 4, 11.4%), *S. lugdunensis* (n = 2, 5.7%), and *S. haemolyticus* (n = 1, 2.9%).

**Table 2:** Distribution of microorganisms in positive vitreous samples from 35 eyes with acute postoperative endophthalmitis

Microorganism	N	%
Gram-positive	31	88.6
*Staphylococcus epidermidis*	17	48.6
*Staphylococcus aureus*	4	11.4
*Staphylococcus lugdunensis*	2	5.7
*Staphylococcus haemolyticus*	1	2.9
*Streptococcus pneumoniae*	2	5.7
*Streptococcus viridans*	3	8.6
*Enterococcus faecalis*	1	2.9
Unspecific gram-positive bacilli	1	2.9
Gram-negative	3	8.6
*Klebsiella oxytoca*	1	2.9
*Enterobacter cloalae*	1	2.9
*Bacillus sp.*	1	2.9
Fungi	1	2.9
*Hormonema* sp.	1	2.9
Total	35	100

The antibiotic resistance profile of the isolated strains is summarized in Table 3. All isolates were susceptible to vancomycin, linezolid, rifampicin, teicoplanin and tigecycline. Most of the tested strains (n = 14/22, 63.6%) were resistant to ciprofloxacin (second-generation fluoroquinolone), many (n = 3/6, 50.0%) were resistant to levofloxacin (third-generation fluoroquinolone), and some (n = 7/20, 35.0%) were resistant to moxifloxacin (fourth-generation fluoroquinolone). One (25.0%) of the 4 strains of *Staphylococcus aureus* was both methicillin-resistant (MRSA) and ceftazidime-resistant, but susceptible to fluoroquinolones.

**Table 3:** Percentage of resistant isolates from eyes with acute postoperative endophthalmitis. Period: 2010–2019

	Ciprofloxacin		Levofloxacin		Moxifloxacin		Oxacillin		Vancomycin		Gentamicin		Ceftazidime		Linezolid	
	n (N)	%R	n (N)	%R	n (N)	%R	n (N)	%R	n (N)	%R	n (N)	%R	n (N)	%R	N (N)	%R
Gram-positive																
*Staphylococcus epidermidis* (n = 17)	13 (14)	93	3 (3)	100	6 (14)	43	17 (17)	100	0 (17)	0	1 (17)	6	17 (17)^†^	100	0 (17)	0
*Staphylococcus aureus* (n = 4)	0 (3)	0	0 (1)	0	0 (3)	0	1 (4)	25	0 (4)	0	0 (4)	0	1 (1)^†^	100	0 (4)	0
Other Coagulase-negative Staphylococci* (n = 3)	1 (3)	33	NT		1 (3)	33	2 (3)	67	0 (3)	0	0 (3)	0	2 (3)^†^	67	0 (3)	0
*Streptococcus pneumoniae* (n = 2)	NT		0 (2)	0	NT		NT		0 (2)	0	NT		NT		NT	
*Streptococcus viridans* (n = 2)	NT		NT		NT		NT		0 (2)	0	NT		NT		NT	
*Enterococcus faecalis* (n = 1)	NT		NT		NT		NT		0 (1)	0	0 (1)	0	NT		0 (1)	0
Gram-negative																
*Klebsiella oxytoca* (n = 1)	0 (1)	0	NT		NT		NT		NT		0 (1)	0	0 (1)	0	NT	
*Enterobacter cloacae* (n = 1)	0 (1)	0	NT		NT		NT		NT		0 (1)	0	0 (1)	0	NT	
Total	14 (22)	64	3 (6)	50	7 (20)	35	20 (24)	83	0 (29)	0	1 (27)	4	20 (23)	87	0 (25)	0

n: number of resistant isolates; N: number of isolates tested; %R: percentage of resistant isolates; NT: not tested; *Staphylococcus lugdunensis and *Staphylococcus haemolyticus;* ^†^ For *Staphylococcus* spp., isolates resistant to oxacillin were considered resistant to cephalosporins

**Fig. 1:**
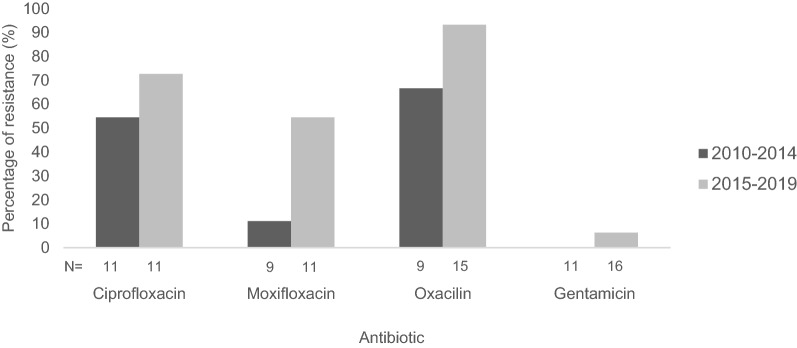
Antimicrobial resistance of bacterial isolates from eyes with acute postoperative endophthalmitis. Comparison of the periods 2010–2014 and 2015–2019

Resistance to 4 of the tested antibiotics increased over time. Thus, comparing the two periods (2010–2014 vs. 2015–2019), an increase was observed for ciprofloxacin (from 6/11 [54.5%] to 8/11 [72.7%], *p* = 0.659), moxifloxacin (from 1/9 [11.1%] to 6/11 [54.5%], *p* = 0.07), oxacillin (from 6/9 [66.7%] to 14/15 [93.3%], *p* = 0.13), and gentamicin (from 0/11 [0%] to 1/16 [6.3%], *p* > 0.999). However, none of these differences reached statistical significance (Fig. 1).

## Discussion

In this 10-year study (2010–2019) conducted at a tertiary referral center within the Brazilian public health care system, we determined the rate of APE based on a sample of 40,491 cataract surgeries. The observed rate (0.126%) is compatible with the rates reported in the literature over the last decades, 0.029 to 0.29% [2–4]. The overall incidence of APE slowly decreased from 0.32% in the 1970s to 0.08% in the 1990s, but then increased to 0.26% in the early 21st century, probably due to wound leakage associated with the adoption of sutureless clear corneal incision [15]. The recent new decrease in APE rates may be explained by better teaching methods [2], the use of fourth-generation fluoroquinolones and the introduction of anterior chamber injection of antibiotics at the end of the surgery [5–7].

The observed culture positivity (71.4%) is within the range (48.0–87.1%) reported over the last years [2–4]. In this study, positivity increased over time, probably due to the adoption of pediatric blood culture bottles in microbiological diagnosis [11]. *Staphylococcus epidermidis* was predominant (n = 17/35, 68.6%) in our study, matching earlier studies showing Gram-positive bacteria to be a frequent contaminant from conjunctiva or eyelid flora [2, 5, 16], but reports from Sweden and South Korea found higher percentages of *Enterococcus faecalis* (20.8–33.3%) than S. *epidermidis * [3, 17]. *E. faecalis* is a natural part of the conjunctiva and eyelid flora. It has been hypothesized that the high incidence of *E. faecalis* in APE cases from Sweden and South Korea is due to the frequent use of fluoroquinolones, which are known to be less effective against this pathogen. However, *E. faecalis* was also predominant in a 12-year study from Taiwan where fluoroquinolones are not routinely used [18]. In our sample, only one case of *E. faecalis* was detected.

Antibiotic resistance may lead to failure of APE prophylaxis and treatment. For example, 1 out of 4 strains of *Staphylococcus aureus* (25.0%) was found to be MRSA. Previous reports have shown that up to 40% of APE-associated *S. aureus* are MRSA, and that MRSA may be associated with multidrug resistance, including fluoroquinolones [19]. We also observed a non-significant increase in resistance against oxacillin, ciprofloxacin and moxifloxacin during the study period. All *Staphylococcus epidermidis* strains were methicillin-resistant, and 6 of 14 (42.8%) were resistant to moxifloxacin. In a study covering the period 2006–2016, Yannuzzi et al. reported methicillin-resistance in 30 (63.8%) of 47 strains of APE-related *S. epidermidis*, but only 29 (34%) of 85 strains of *S. epidermidis* were susceptible to moxifloxacin [20]. In a 22-year study from the Bascom Palmer Eye Institute (USA) evaluating fluoroquinolone non-susceptibility among APE-related coagulase-negative *Staphylococcus*, non-susceptibility to moxifloxacin increased from 22% (1995–1999) to 57% (2010–2016) (*p* = 0.003) [21].

It is hoped the recent improvements in prophylaxis, surgical technique and treatment will reduce the incidence of APE to < 0.1% [22]. To do so, surgical teams must adopt the best aseptic and prophylactic practices available and carefully monitor perioperative risk factors.

As for asepsis, preoperative povidone-iodine has proved to be the only measure supported by level II evidence capable of reducing APE rates. In addition, it is inexpensive, easy to use and associated with very low rates of complications [23–25]. The effective concentration and exposure time vary between countries and studies, but povidone-iodine is usually administered preoperatively 0.25-5% for 30 seconds to 3 min [23–25]. Some authors have suggested administering it after surgery in order to reduce the flora of the conjunctiva. However, an Iranian randomized clinical trial found similar colony counts for patients receiving povidone-iodine post-operatively and patients treated with sub-conjunctival antibiotics at the end of the surgery [26].

Changeable risk factors are related to surgical techniques (administration of 2% lidocaine gel before povidone-iodine drops, clear corneal incision, extracapsular surgery, surgeon skill, silicone intraocular lens, surgical complications, wound leakage) and hospital stay [3, 8, 27–30].

The prophylactic use of IC antibiotics, especially cefuroxime, vancomycin and moxifloxacin, has been shown to reduce the risk of APE. Cefuroxime (1 mg/0.1mL), a second-generation cephalosporin, reduced risk from 0.296 to 0.062% in a randomized controlled study by the European Society of Cataract and Refractive Surgeons (ESCRS) [5]. Moxifloxacin (0.1–1 mg/0.1mL), a fourth-generation fluoroquinolone, was associated with an average APE rate of 0.015% in a recent meta-analysis [31] and is commercially available in some countries.

At our institution (a public tertiary referral center), all surgical procedures were performed by residents and fellows, potentially increasing the risk of surgical complications such as posterior capsule disruption and vitreous loss. Nevertheless, adequate preoperative skin surface asepsis was routinely performed using 10% povidone-iodine, in addition to instillation of 5% povidone-iodine eye drops prior to draping. Factors associated with surgeon training are not changeable, but other measures could be implemented to reduce APE rates at our institution. Moreover, taken together, the increased number of surgeons using IC antibiotics (a recently introduced recommendation based on the findings of this study) and the decrease in APE rates deserve further attention from our research teams.

On the other hand, the efficacy of IC antibiotics has been questioned by authors observing low APE rates (0.062%) even without this practice, similar to those reported in ESCRS Study [5]. In a prospective comparative interventional cohort study, following more than 15,000 eyes operated from 2006 to 2010, the APE rate of the group without IC cefuroxime was 0.16%, which is similar to that of the IC cefuroxime group (0.11%) operated from 2010 to 2012 (*p* = 0.57) [32]. Furthermore, antibiotics for IC use (which are still not commercially available in many countries, including Brazil) should be properly prepared by the institutional pharmacy: when surgeons have to prepare the antibiotic themselves, there is an additional risk of dilution error and contamination, thus overdose and ocular toxicity.

The limitations of this study include lack of information on visual outcomes and risk factors, and the small size of each subsample. Nevertheless, covering a relatively extensive period, we present highly relevant data on APE incidence, microbiological spectrum and antibiotic resistance needed to effectively introduce novel prophylactic measures, such as IC antibiotics.

## Conclusions

The microbial spectrum of our APE patients was similar to that of previous studies, showing the current empirical treatment with vancomycin and ceftazidime to be adequate. Based on our data, our institution might consider adopting IC antibiotic therapy to reduce APE rates. However, the observed trend toward growing moxifloxacin and ciprofloxacin resistance should be monitored closely.

## Data Availability

The dataset used during the current study is available from the corresponding author on reasonable request.

## Abbreviations

APE: Acute postoperative endophthalmitis
MRSA: Methicillin-resistant *Staphylococcus aureus*
IC: Intracameral
